# Keeping a lid on calcium uptake

**DOI:** 10.7554/eLife.17293

**Published:** 2016-06-03

**Authors:** Vivek Garg, Yuriy Kirichok

**Affiliations:** Department of Physiology, University of California, San Francisco, San Francisco, United Statesvivek.garg@ucsf.edu; Department of Physiology, University of California, San Francisco, San Francisco, United Statesyuriy.kirichok@ucsf.edu

**Keywords:** calcium transport, MCU, EMRE, mitochondria, ion channel, Human

## Abstract

Biochemical assays reveal how three proteins fit together to form the channel that controls the flow of calcium ions into mitochondria.

**Related research article** Tsai MF, Phillips CB, Ranaghan M, Tsai CW, Wu Y, Williams C, Miller C. 2016. Dual functions of a small regulatory subunit in the mitochondrial calcium uniporter complex. *eLife* **5**:e15545. doi: 10.7554/eLife.15545

**Image** Schematic showing calcium ions (gray) flowing through an open ion channel

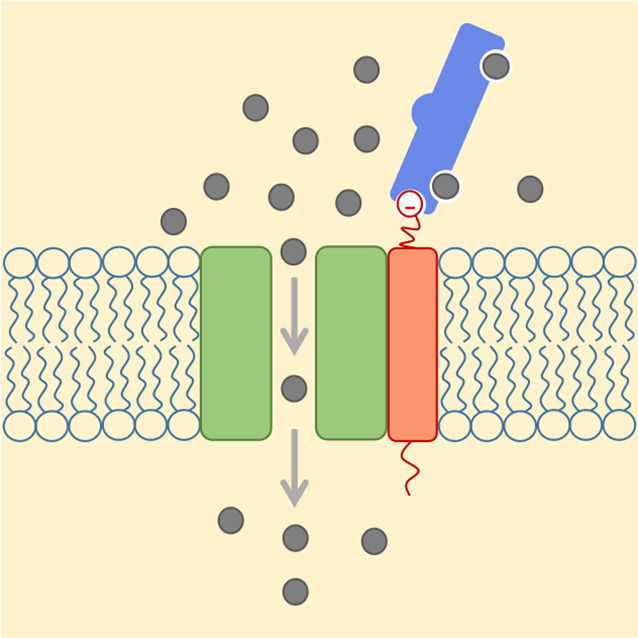

Mitochondria are often referred to as the “powerhouses” of eukaryotic cells because they supply most of the energy that the cells need. In the 1960s it was discovered that active mitochondria, when isolated from the cell and studied “in a test-tube”, accumulate large quantities of calcium ions (Ca^2+^). However, the importance of this phenomenon was not immediately clear. Later, in the 1990s, it was revealed that mitochondria inside eukaryotic cells also take up Ca^2+^ ions (Rizzuto et al., 1998).

The uptake of Ca^2+^ by mitochondria stimulates certain enzymes to regulate energy production in order to match the cell’s activity. However, if too much Ca^2+^ enters, the mitochondria can malfunction which often kills the cell. The uptake of Ca^2+^ by mitochondria must therefore be tightly controlled. Now, in eLife, Christopher Miller and colleagues at Brandeis University – including Ming-Feng Tsai and Charles Phillips as joint first authors – report how this control might be achieved (Tsai et al., 2016).

Each mitochondrion has an inner membrane and an outer membrane. Small molecules and ions (including Ca^2+^ ions) can pass freely through the outer membrane, but not the inner one. The transport of Ca^2+^ through the inner membrane depends on an ion channel called the “mitochondrial Ca^2+^ uniporter” (or MCU channel for short). This channel is the most selective Ca^2+^ channel currently known (Kirichok et al., 2004).

The MCU channel is actually a protein complex made from multiple subunits. The Ca^2+^ ions pass through a pore-forming subunit (Baughman et al., 2011; De Stefani et al., 2011) that spans the inner membrane and is surrounded by five other subunits. These other subunits regulate the pore-forming subunit, but how they do this and how they are all assembled into the channel complex are still topics of active debate.

The pore-forming subunit plus two of the five regulatory subunits (proteins named EMRE and MICU1) form what can be referred to as the “core functional unit of the MCU” (Perocchi et al., 2010; Sancak et al., 2013). This stripped-down version of the complex acts much like the full channel and can be used to explain how mitochondria take up Ca^2+^. Tsai, Phillips and colleagues used biochemical assays to determine how these three subunits fit together within the core functional unit. They demonstrated that EMRE interacts with the pore-forming subunit via domains that span the inner membrane. They also found that the subunits could not form a working channel without this interaction. Furthermore, they showed that MICU1 binds to EMRE at the outer surface of the inner mitochondrial membrane (Figure 1).

**Figure 1.**
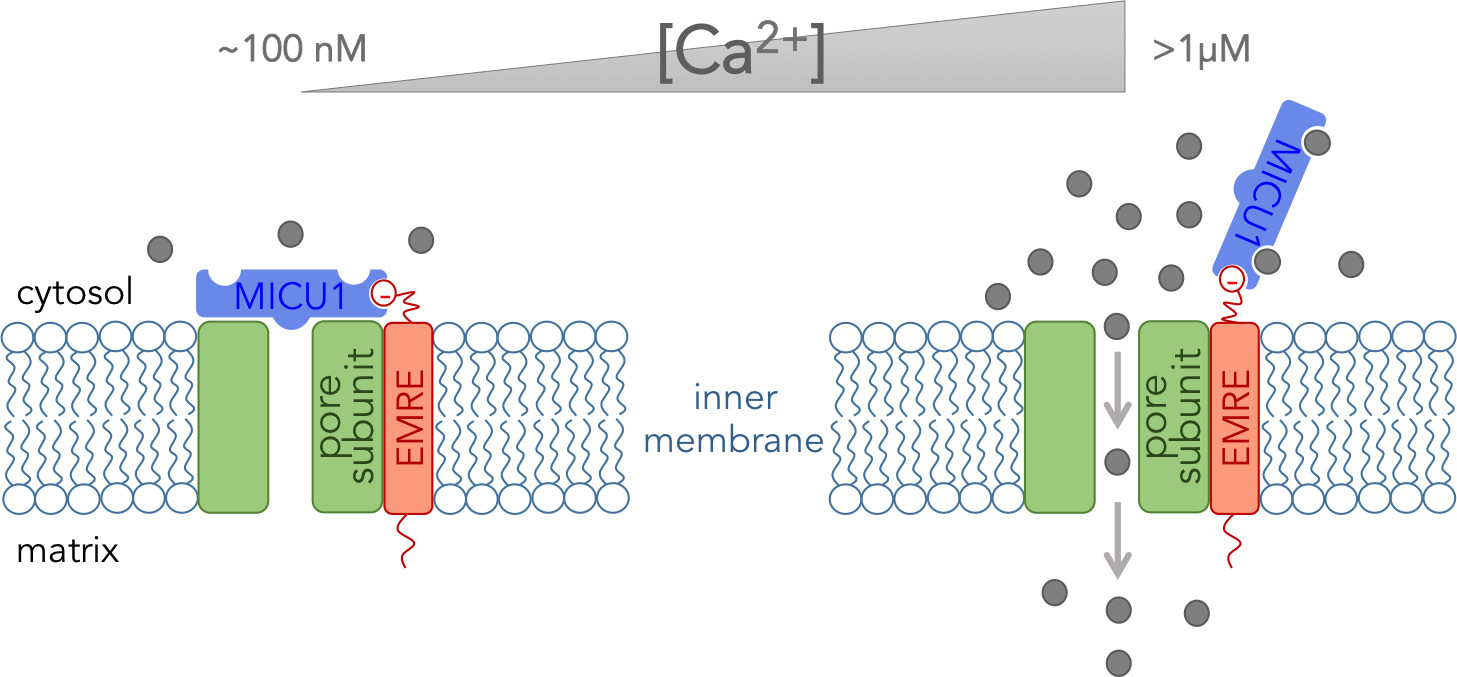
The core functional unit of the MCU channel complex. The core functional unit spans the inner membrane of a mitochondrion and consists of three subunits: the pore-forming subunit (green), MICU1 (blue) and EMRE (red). Tsai, Phillips and colleagues show that the pore-forming subunit and EMRE interact within the inner membrane via their transmembrane domains. They also show that a negatively charged domain of EMRE (red circle) anchors MICU1 to the cytosolic face of the inner mitochondrial membrane. The concentration of calcium ions ([Ca^2+^]) in the cytosol of a resting cell is typically about ~100 nM (left). At this concentration, MICU1 does not bind to Ca^2+^ ions (gray circles), and MICU1 blocks the pore to prevent the flow of Ca^2+^ ions. In contrast, when the concentration of Ca^2+^ in the cytosol is elevated (right), MICU1 binds to two Ca^2+^ ions and dissociates from the pore. This allows Ca^2+^ ions to flow into the mitochondria (gray arrows).

Combined with relevant data from other groups (Mallilankaraman et al., 2012; Csordás et al., 2013; Patron et al., 2014), the results of Tsai, Phillips and colleagues provide a glimpse of how the MCU channel complex might work at the molecular level. EMRE anchors MICU1 near the pore-forming subunit, and MICU1 then blocks the pore when the Ca^2+^concentration in the cytosol is at its resting level. This stops Ca^2+^ ions from flowing into the mitochondria. However, when the Ca^2+^ concentration in the cytosol increases, Ca^2+^ ions bind to MICU1and cause it to dissociate from the pore to allow other Ca^2+^ ions to pass through (Figure 1). Thus MICU1 serves as a Ca^2+^-sensitive “lid” on the MCU channel complex, which closes and opens the channel in response to changes in the Ca^2+^ concentration in the cytosol. Notably, the pore-forming subunit cannot work without EMRE (Sancak et al., 2013). Thus it might be EMRE, and not the pore forming subunit, that controls how many of the MCU channels are active in various tissues.

Now that we know how the MCU core functional unit is assembled, the stage is set to explore how the structure of the MCU channel relates to its function. This will bring us closer to understanding the phenomenon of Ca^2+^ uptake by mitochondria and how it could be affected via drugs to control energy production in cells and cell death.
